# Case of Ulceration and Perforation of the Aorta, with Large Polypi Blocking up Its Caliber

**Published:** 1844-10-05

**Authors:** Geo. L. Upshur


### THE MEDICAL EXAMINER,

### And Record of Medical Science.

Vol. VII.] PHILADELPHIA, SATURDAY, OCTOBER 5, 1844. [No. 20.

### CASE OF ULCERATION AND PERFORATION OF THE AORTA, WITH LARGE POLYPI BLOCKING UP ITS CALIBER.

By Geo. L. Upshur, M. D.

To the Editor of the Medical Examiner.

I transmit below the history of an interesting case, as reported to me by Dr. Plummer, of the Navy, through Commander Sands, of the U. S. ship of War Falmouth. If you think, with me, that it is calculated to throw any light upon the diagnosis and treatment of an exceedingly grave and rare disease, you are at liberty to give it to the medical world through the columns of your valuable Journal. I have appended some remarks to the report.

It may be proper to state, as explanatory of some of the passages in the letters of Drs. Plummer and Bee, that the late Purser Upshur, prior to his entering the Navy, had been a practitioner of medicine for six or seven years.

Geo. L. Upshur, M. D.

"*U. S. Ship Falmouth,*

At Sea, Sept. 7th, 1844.

*Sir:*—In accordance with your wishes, expressed in your note dated on the 6th inst., I herewith transmit a short, and, at the same time, as correct a statement as it is in my power to give, of the symptoms, treatment, &c., of the case of the late Arthur W. Upshur, Esq., Purser, for the information of his medical friends.

I also enclose herewith, for the same purpose, a paper on the same subject, from Dr. E. J. Bee, the Assistant Surgeon of this vessel, which should, and, I fondly hope, will, give great satisfaction to the friends of the deceased.

Mr. U., on leaving Old Point, had facial neuralgia, for which he took, then, and had been in the habit of taking for the same malady, 1-6th of a grain of acetate of morphia almost every night. About that time, he also took B. Mass, et Ext. Col. Comp. *aa.* gr. v., and, after its operation, he complained no more of the neuralgia.

During our stay at Vera Cruz, there was continual thunder and lightning—showers of rain at night succeeded by great heat during the day; Fah. thermometer ranging between 82° and 85° in the shade. From first to last, however, there was nothing simulating yellow fever in his case, although he must have been very much exposed, with other officers, in his visit up to the city under a broiling tropical sun.

On the 23d of August, he remarked, that, for a long time previous, he had had great difficulty of breathing on ascending heights, with an occasionally intermitting pulse, and at that moment I felt his pulse, and found it intermitting once every minute, full also, and rather slow. As it was very warm in his state-room, (thermometer 80°) he slept upon deck, and caught cold, on the night of the 25th. On the 26th he complained of pain in the left side, with a dry, tickling cough, for which venesection was proposed, but he would not submit to it, as he believed it to be merely an attack of asthma, or else a metastasis of the neuralgia on which the pain in the side had supervened; for he said also, that he had had the pain in the side ever since four days out from the Capes of Virginia, just about the time of the disappearance of the neuralgia; so that the reasons for his belief were quite plausible. Up to this time, 26th of August, he had not said anything of the pain in the side, either to Dr. Bee or to myself, otherwise I should have endeavored to persuade him to be bled q. s. For this he was cupped, which relieved him somewhat, but on the evening of the 27th he was obliged to have applied an Emp. Ves. which drew very well, and for the time relieved the pain entirely. He also took, that evening, Hyd. Chlor. Mit. gr. x. P. opii. gr. ij.—rested tolerably well.

28th, Evening. Took B. Mass. gr. v.; P. opii gr. ij.—passed a bad night, blister discharged well.

29th, 9 A. M. We persuaded him to have ℥xiv. of blood taken away, which was not at all buffy; bore it tolerably well, and breathed much freer until towards evening, when he took Hyd. Chlor. Mit. gr. x.; P. opii gr. ij.

30th, Morning. Very restless night—in the morning, complained of pain in the chest, shooting back from the lower end of the sternum to the spine, and of pain opposite the 6th dorsal vertebra, which, however, he said was relieved by pressure instead of being increased, as is the case where spinal irritation exists; respiration entirely abdominal; deglutition difficult, and complained of a scalding along the whole tract of the œsophagus; tongue quite furred; pulse 100, and very soft; a tremulous motion of the right foot and leg; difficult micturition; pain at the top of the sternum with a suffocating sensation. Prescribed dry cups to the sternum, and emp. ves. to sternum and lower part of the trachea; syr. orgeat. flaxseed tea, and sub-acid drinks; but what he mostly relied on was the acet. morph. in gum water.

31st. Passed a very restless night; countenance more haggard; pulse 100, and fluttering; aq. camph. p. r. n., acet. morph. gr. ij. in Muc. G. Arabic ℥viij-s. ℥ss. every four hours, at his own particular request, as he said that nothing else gave him any relief. Flaxseed tea; c. cups, No. 9, to spine and right chest; cups to spine and right chest, No. 6; dry cups to spine and epigastrium.

Sept. 1st. Cut cups No. 2 to nucha; dry cups, No. 9 to back of neck, between shoulders, epigastrium, and also over cardiac region; accordingly as the pain shifted, there the cups were applied; morphine and mucilage as usual; sweetened mucilage; sinapisms over various parts of the chest; took, at his own request, Ol. Ricini ℥ij., with ess. of peppermint, but immediately rejected it; stimulating enema to relieve tympanitis; great difficulty of swallowing; pulse 120; warm spiced brandy to epigastrium; lin. ammon. to spine.

Sept. 2nd. In the night emp. ves. 11 in. by 3 in. was applied over the spine between the shoulders, and it drew this morning, with some relief. Took 1-4th gr. Ext. Belladonna; at 6 A. M. was easier; pulse 120, but soft; tongue soft and moist; drank a cup of tea through a quill; became worse again about 9 A. M., and died at half past 10 A. M., retaining his senses perfectly up to the last moment.

*Autopsy twenty hours after death.*

Strong evidences of putrefaction, viz: putrid odor, and a bluish discolouration about the hypogastric and iliac regions; found the stomach natural, except a few small gray patches, resulting probably from gastric inflammation; the posterior part much injected with blood, but cadaveric. The blood could be pressed out of the vessels, which is not the case with an inflamed stomach.

*Spleen.* Twice the natural size; no lesions; blue at its apex; no softer than a healthy one.

*Liver.* Healthy, but very much enlarged, extending below the false ribs, and partly over the spleen; acini enlarged; gall bladder healthy; no calculi. *Kidneys*, healthy.

*Heart.* Has been no inflammation of the pericardium; endocardium presents no indication of inflammation either acute or chronic; valves healthy; left ventricle slightly dilated, and its walls, perhaps, a little hypertrophied. The condition of the heart (organic) does not explain the cause of the pulse intermitting.

*Lungs*—Right. Pleura adherent throughout to the walls of the chest; very much congested in the upper lobe, but no pus, nor hepatization; middle lobe, hepatized, yellowish, and in apex an abscess the size of a hen's egg, partly filled with strumous blood and pus. The smaller bronchial tubes and vessels subtend the cavity. Some of them simulate in appearance the chordæ tendinæ of the heart; a few tubercles in apex in different stages of developement. The lowest lobe very much consolidated, and discharged a considerable quantity of strumous matter on pressure.

*Left lung.* The adhesions of the superior lobe exist in patches only; inferior one adhesive entirely. Very emphysematous along its anterior margin; air vesicles much distended; presented on its surface an appearance very similar to the blotches on the skin of a poisoned person, only instead of being red they were a light yellow; interior too much congested to examine the vesicles; considerable bloody greyish spuma issued on pressure.

The trachea, larynx, and œsophagus, healthy.

Ascending aorta, natural, also the arch, but in the descending aorta, nearly opposite the breast, and a little below, found an ulceration opening through the coats of the artery, nearly round, and in size about that of an English shilling. Around this opening are several smaller ulcers, about the size of millet seed, or the point of a pin. Found in the aorta, about an inch below the opening, a small clot of blood, about the size of a shellbark hickory-nut with the hull on it. There was, also, protruding from the orifice into the aorta, the pointed end of a coagulum of blood. On pulling it through the opening, it was found to be four inches long, and in shape something like a gimlet handle. Presented the appearance of polypi of the heart. A long, bloody, oblong sack, extending nearly from the diaphragm to the kidneys, was found—in the abdomen the aneurism was between the spine and peritoneum. The abdominal aorta and intestines appeared healthy, but, from the somewhat hasty manner in which we were obliged to make the examination, we did not pay much attention to the abdominal viscera.

To the best of my ability, I have here endeavoured to condense this statement as much as possible, (but it has been extended, perhaps, to a tiresome length,) and submit it, with all its imperfections upon its head, to the supervision of the medical friends, trusting that they will believe me when I say, that I exerted myself to do all that could be done on board ship, in so hopeless a case as it has been proved to be by the result of the post-mortem examination.

I am, sir, sincerely,
Your much obliged, &c.
J. W. Plummer, Suregon.

J. R. Sands, Esq., Commander.

The following are extracts from Dr. Bee's letter to Dr. Plummer, and contain a very satisfactory account of an examination of the chest of the deceased, together with a history of some of some of his symptoms, before he was put upon the sick list of the ship:

"*U. S. Ship Falmouth, Sept. 6th*, 1844.

"Dear Doctor,

"In accordance with your request, I give below some of the conversations which took place between the late Purser and myself, touching his complaint as existing immediately after we sailed; and also the result of a very imperfect examination of his chest.

The fourth day at sea, while smoking with the late Purser, I alluded to his dyspnœa, which I had observed ever since I was acquainted with him, being about six weeks. I asked him if it was not asthma. He was not aware of being an asthmatic, but supposed that the dyspnœa depended upon his heart being diseased. He told me, that physicians on shore pronounced his heart hypertrophied. (Post-mortem has not verified this opinion—the change from a healthy heart being too slight for any ausculation to detect.)

At his request, I examined his chest, (but you are aware that an accurate examination cannot be made on board ship,) and found that there was no vesicular murmur in the left lung—left side of chest fuller than right—intercostal spaces prominent, particularly upon inspiration—sibilant rhoncus—vibration of the voice, felt by the hand being laid on the left chest, very strong; percussion louder in the left than in the right lung. Compared only the intensity of sound between two sides of the chest—too much noise aboard ship to practice percussion. *Right lung.* Vesicular respiration hard in the upper lobe—lower lobes doubtful; bronchial, throughout the whole right lung; lower lobe, tubal; bronchophony, and bronchial resonance in middle and lower lobes. Sound of voice through middle and lower lobes disagreeable to the ear. In percussion, too much noise to distinguish between dull and flat sounds, and therefore did not practice it. I examined his heart, but the sounds were natural, as far as I could distinguish them.

The opinion given him immediately after the examination, was as follows: Emphysema of the left lung--chronic pneumonia of the middle and lower lobes of the right, with hepatization, but whether second or third stage could not say, from not being able to mark the finer sounds. Heart I considered healthy, with allowance for ship examination. He told me his pulse frequently intermitted, but I believe I never observed it. He appeared satisfied that he had emphysema of the left lung. His right lung he thought healthy, except some slight consolidation resulting from a very bad cold he had in Norfolk.

The neuralgic affection he generally doctored himself, although, as a matter of form, he asked my opinion of the remedies, and permission to get them of the steward. These were principally laxatives, counterirritants, and anodynes. His appetite was good—also his digestion—complained of no pain nor difficulty of swallowing—was somewhat costive, for which he prescribed for himself seidlitz powders. He complained of nothing but wheezing and shortness of breath after the neuralgic affection left his face, when the wheezing became worse.

He went ashore at Vera Cruz—came aboard very much fatigued, sunburnt, and complaining of headache. Said he did not want any medicine, but as soon as he had a good night's rest, would be as well as ever. After we sailed from Vera Cruz, he slept one night till late in the quarter boat, and was somewhat chilly when he got out and went below. It was then, he supposed, he took his cold. He prescribed for himself, the evening before he came on the list, a blue pill, followed in the morning with a seidlitz powder. * * * *

I never heard him complain of pain in his breast, until after he took this cold. This pain increased with his sickness, and simulated spinal irritation so much that he was cut cupped, dry cupped, and finally blistered. His back was tender on pressure. Cupping always relieved him. * * *

Yours, respectfully,

Dr. J. W. Plummer, E. J. Bee.
U. S. Ship Falmouth.

*Remarks.*—There are few cases on record presenting to the medical man more points of interest than that detailed above. I had known the deceased for a number of years prior to his death. He was stout, fleshy, of low stature, and of a decidedly strumous diathesis. About three yeears ago, he had a severe attack of remittent fever, which scarcely abated before acute articular rheumatism supervened, from which he did not recover for several weeks. Since then he constantly suffered more or less with dyspnœa, and a dry, hacking cough, accompanied by shooting pains about the sides of the thorax, which simulated spinal irritation. These symptoms, however, were never severe enough to confine him to bed, or even to the house. Some time during the latter part of last June, he took cold, which gave rise to more cough than usual, and some fever. About that time, at his request, I examined his chest, and found his lungs nearly as described by Dr. Bee, except that the morbid sounds were not so well marked in the right lung, and there was altogether an absence of the tubal respiration in the lower lobe. The action of the heart was rather stronger than natural, with a roughness of the first sound, and I told him I thought there was some hypertrophy.

The autopsy showed extensive disease of the lungs, while the heart was comparatively healthy, verifying completely the accuracy of Dr. Bee's diagnosis. The most important lesions, however, were found in the descending aorta, which was, undoubtedly, the primitive seat of disease, the lungs being secondarily involved.

It becomes a matter of interest to inquire, how long prior to death had ulceration and perforation of the aorta existed? From the history of the case, it is probable, that aortitis was co-existent with the articular rheumatism alluded to above. The inflammation subsequently became chronic, giving rise to the ulcerations and fibrinous deposites exhibited by the autopsy. It has long since been demonstrated, by Bouillaud and others, that inflammation of the internal membrane of the heart is present in a majority of cases of articular rheumatism, and, as the internal tunic of the aorta is merely a continuation of the endocardium, it is fair to presume that it may suffer similar lesions during the course of that affection. When we take into consideration the extent of the opening, the size of the clots, the emphysema, hepatization, and tuberculous condition of the lungs, together with the increased size of the liver and spleen, we are almost forced to the conclusion, that ulceration of the artery commenced at a remote period, while the perforation existed for some days, at least, prior to death. On the other hand it would seem, that obstructions so great, offered for any length of time to the free flow of blood from the heart, would have been productive of more hypertrophy and dilatation of the left ventricle than are reported to have existed. It may be, however, that the fibrinous clots did not attain the size described by Dr. Plummer, until a very short time before death.

The existence of the "large, oblong, bloody sac, extending nearly from the diaphragm to the kidneys," is another interesting point in this case. It is to be regretted, however, that the report does not locate more definitely the connections of this sac. We are at a loss to determine, whether it started from near the diaphragm *in the thorax*, or *in the abdomen*—whether it was merely a blind pouch filled with blood, or, the flow of blood being obstructed in the artery, it formed a collateral channel for the circulation, by connecting with the aorta above and below the obstruction. If it was not spoken of in the report as an "aneurism," I should be inclined to think that it was a closed sack, formed by effusion of blood at the moment of perforation.

There seems to have been an intimate relation between the neuralgia and the disease of the aorta, or of the lungs. The report says, that as soon as the neuralgia ceased to show itself in the face, the cough and dyspnœa became more troublesome. Upon what was this dependent? I have several times seen severe facial neuralgia during the course of pulmonary diseases, especially in pneumonia, occurring in anemic persons. I leave to wiser heads the task of explaining the phenomenon.

*Norfolk*, (*Va.*) Sept. 26th, 1844.

